# When measuring leads to better outcome in cardiogenic shock

**DOI:** 10.47487/apcyccv.v1i4.102

**Published:** 2020-12-31

**Authors:** Holger Thiele

**Affiliations:** 1 Heart Center Leipzig at University of Leipzig, Leipzig, Germany Universität Leipzig Heart Center Leipzig University of Leipzig Leipzig Germany

Current features and mortality risk factors in cardiogenic shock due to myocardial infarction in a Latin-American hospital (p. 206). 

Cardiogenic shock (CS) is still the most important cause of death in patients admitted with acute myocardial infarction. The randomized SHOCK (Should we emergently revascularize Occluded Coronaries for cardiogenic shock) trial set the basis for an early invasive management of these patients ^1^, with subsequent early revascularization. This strategy has dramatically improved outcome and reduced in-hospital mortality of CS patients from the former 70-80% to nowadays 40-50% ^2^.

Currently, only few large-scale randomized trials have been performed in the CS setting including the above-mentioned SHOCK trial in 1999 ^1^, the TRIUMPH trial ^3^, the IABP-SHOCK II trial ^4^^-^^6^, and also the CULPRIT-SHOCK trial ^(7, 8)^. Accordingly, only few measures rely on strong clinical evidence in the treatment of CS ^9^^,^^10^.

If insufficient evidence is available and mortality still high, evidence from observational data is important. Even more important in clinical practice is to measure the outcome of acute coronary syndromes and the complications including CS. Only by measuring outcome, measures can be implemented to improve outcome. As such it can only be supported to see the publication of the National Cardiovascular Institute INCOR. The in-hospital mortality of 70% of this heterogenous group of CS patients in this reference center shows the still very high mortality in CS. Interestingly, still the majority of patients is treated by intraaortic balloon pumping where the evidence does not support to use this device ^4^^-^^6^. On the other hand evidence for active mechanical circulatory support is also limited and currently no larger randomized controlled trial has shown any mortality benefit for active mechanical circulatory support ^9^. 

In general, the pathophysiology of CS is complex and is characterized by a profound depression of myocardial contractility, resulting in a vicious spiral of reduced cardiac output, low blood pressure, further coronary ischemia, and subsequent reduction in contractility and cardiac output. In addition, CS is heterogenous and therefore scores to assess CS mortality are important. It is interesting to see that both the IABP-SHOCK II and the SCAI shock definition worked well in the current registry ^11^^,^^12^.

The authors should be congratulated to put this Peruvian registry together. More efforts should be directed towards CS registries and a higher number of patients will help to define the best treatment strategies and improved outcome in CS.
